# Effects on Serum Fractalkine by Diet and Omega-3 Fatty Acid Intervention: Relation to Clinical Outcome

**DOI:** 10.1155/2015/373070

**Published:** 2015-02-05

**Authors:** Kristian Laake, Ingebjørg Seljeflot, Morten Wang Fagerland, Ida Unhammer Njerve, Harald Arnesen, Svein Solheim

**Affiliations:** ^1^Center for Clinical Heart Research, Department of Cardiology, Oslo University Hospital, Ullevål, Postboks 4956 Nydalen, 0424 Oslo, Norway; ^2^Faculty of Medicine, University of Oslo, Postboks 1078 Blindern, 0316 Oslo, Norway; ^3^Center for Heart Failure Research, Institute for Experimental Medical Research, Oslo University Hospital, Ullevål, Kirkeveien 166, 0407 Oslo, Norway; ^4^Oslo Centre for Biostatistics and Epidemiology, Research Support Services, Oslo University Hospital, Postboks 4950 Nydalen, 0424 Oslo, Norway

## Abstract

*Introduction*. Fractalkine is a chemokine associated with atherosclerosis. Increased serum levels have been reported in unstable coronary artery disease (CAD) and to predict mortality in heart failure. Mediterranean-like diet and omega-3 fatty acids (n3-PUFA) have documented cardioprotective and anti-inflammatory effects. We have investigated the effect of Mediterranean-like dietary counseling and n-3 PUFA on serum fractalkine in an elderly population and its ability to predict cardiovascular disease (CVD). *Materials and Methods*. 563 men (age 64–75 yrs) at high risk of CAD were randomized into a 2 × 2 factorial designed trial for 3-year dietary counseling and/or n-3 PUFA supplementation (2.4 g/d). Circulating levels of fractalkine were measured at baseline and at end of study. Clinical events were recorded after 3 years. *Results*. Fractalkine levels were significantly reduced in all groups from baseline to 3 years (*P* < 0.001, all), but without between-group differences in changes. Fractalkine levels at baseline were not predictive for CVD events (*n* = 68) or total mortality. Lower fractalkine levels were observed in smokers (*P* = 0.019). *Conclusions*. Reduced levels of fractalkine from baseline to 3 years were observed, however, without any influence of Mediterranean-like diet or n-3 PUFA supplementation. Fractalkine levels at baseline were not predictive for later CVD events.

## 1. Introduction

Atherosclerosis, long time viewed as a cholesterol storage disease, is today considered to be an inflammatory process involving cytokines and chemokines and the infiltration of monocytes and T-cells into the vessel wall [1]. Fractalkine (CX3CL1) is a chemokine expressed by multiple cells in the body with a CX3C motif. Its receptor (CX3CR1) is expressed on various proinflammatory cells such as monocytes, T-cells, and natural killer cells [2, 3]. Fractalkine has a dual function as it exists both as a transmembrane protein implicated in cell adhesion and migration and in a cleaved form acting as a chemoattractant, both contributing to vascular inflammation and injury [4, 5]. Circulating levels of fractalkine/CX3CL1 have been shown to correlate with disease activity in a broad range of inflammatory diseases [6]. The possible role of fractalkine in atherosclerosis is mainly shown in animal studies [5, 7, 8]. Staining of human coronary arteries with atherosclerotic plaques has shown upregulation of fractalkine [9], and it has been demonstrated to have proliferative and antiapoptotic effects on primary smooth muscle cells, considered an important factor in plaque stability and vessel stenosis [10]. A genetic polymorphism in the fractalkine receptor has been associated with reduced risk for developing coronary artery disease (CAD) [11] and unstable CAD patients have shown increased plasma levels of fractalkine [12]. Richter et al. found fractalkine to be an independent predictor of mortality in a group of elderly patients with advanced heart failure (HF) [13]. In recent studies, circulating fractalkine was shown to correlate significantly with serum triglycerides and to be a predictor of the development of metabolic syndrome [14], but its association with type 2 diabetes mellitus is ambiguous [15, 16].

Mediterranean-like diet and omega-3 polyunsaturated fatty acids (n3-PUFA) have, with somewhat conflicting results, shown beneficial influence on cardiovascular disease (CVD) in large scale clinical trials [17–21]. Studies on mechanisms behind the effects have revealed both anti-inflammatory and antithrombogenic effects in addition to plaque stabilizing properties [22–25]. Very limited knowledge on the effects of n-3 PUFA and Mediterranean-like diet on fractalkine levels exists [26]. The aim of the present study was therefore to evaluate the effect on circulating fractalkine levels after 3-year diet counselling or n-3 PUFA supplementation in an elderly population at high risk for CAD. Furthermore we wanted to explore the predictive value of fractalkine on CVD events and mortality and also to study any relation to disease entities in this population.

## 2. Materials and Methods

### 2.1. Participants

The Diet and Omega-3 Intervention Trial on Atherosclerosis (DOIT) [27] creates the basis for the current investigation. DOIT was designed as a long-term follow-up of the participants in the diet and antismoking intervention of the Oslo Study [28] undertaken in 1972–77. Of the 1232 men with hyperlipidemia and high risk of CVD, 563 survivors between the age of 65 and age of 75 were enrolled into the trial starting in 1997. The study was carried out in compliance with the Helsinki Declaration and approved by the Regional Ethics Committee. All subjects gave their written informed consent to participate (ClinicalTrials.gov, NCT00764010).

### 2.2. Study Design

The present study is a substudy of the DOIT trial that has previously been reported in detail [27]. In short, the study was a prospective randomized trial evaluating the effect of a 3-year intervention with Mediterranean-like dietary counseling, n-3 PUFA supplementation (2.4 g/d), or both, on the progression of atherosclerosis in a high-risk population of elderly men. It had a 2 × 2 factorial design and was placebo controlled for the n-3 PUFA capsules. The dietary counselling was adapted individually by a clinical nutritionist with the recommendation to replace saturated with monosaturated fats. This was to be accomplished by increased intake of vegetable oils and soft margarines (rapeseed oil, olive oil, and sunflower oil), vegetables, fruits, and fish with a simultaneous decrease in the use of meat and fat from animal sources. Special oil and margarine (VITA margarine; Mills DA, Norway) were specifically supplied. The n-3 PUFA capsules (Pikasol, provided by Lube, Denmark) contained 35% eicosapentaenoic acid (EPA) (C20:5n-3), 20% docosahexaenoic acid (DHA) (C22:6n-3), and 3.5 mg/g tocopherols in order to prevent fatty acid peroxidation. The placebo capsules contained 56% linoleic acid (C18:2n-6), 32% oleic acid (C18:1n-9), 10% palmitic acid (C16:0), and 4 mg/g tocopherols. Compliance, evaluated by serum levels of fatty acids and food frequency questionnaires, was recorded at baseline and at 36 months. The participants visited or had telephone contact with the nutritionist every 6 months with adherence to diet evaluated by nutrient patterns reported in questionnaires [27]. Hypertension was defined as systolic blood pressure >140 and/or diastolic blood pressure >90 mmHg and diabetes as manifest diabetes and/or fasting glucose >7 mmol/L and metabolic syndrome according to the Adult Treatment Panel III (ATP-III) definitions [29]. Smokers were defined as current smokers.

### 2.3. Laboratory Methods

Blood samples were obtained in fasting condition (>10 h) by standard venipuncture before daily intake of medication between 08:00 and 10:00 a.m. Serum was separated by centrifugation within 1 h at 2500 ×g for 10 min and kept stored at −80°C until determination of fractalkine and serum fatty acids. Fractalkine was quantified by commercially available enzyme-linked immunosorbent assay (ELISA) kits from R&D Systems (Abingdon, Oxon, UK). Coefficient of variation was 7.8% in our laboratory. Serum lipids were determined by conventional enzymatic methods. All samples were analyzed in the same run to avoid bias due to assay variability.

### 2.4. Statistics

The results are presented as median values unless stated otherwise. Nonparametric statistics were used and the results were calculated according to the original 2 × 2 factorial design. To compare the intervention groups at baseline, Mann-Whitney *U* test was used for continuous variables whereas *χ* ^2^ test was applied for the categorical variables. Between-group differences in relative changes were evaluated by Mann-Whitney *U* test and intragroup changes from baseline to 36 months by Wilcoxon test. Analyses of correlation were performed with Spearman rho. Survival curves were created using the Kaplan-Meier method. Cox regression analysis was used to estimate the predictive value of quartiles of fractalkine on CVDs and mortality. We checked for interaction between smoking and fractalkine. The results are given as hazard ratios (HRs) and 95% confidence intervals (CI). A two-tailed value of *P* ≤ 0.05 was considered statistically significant. The statistical analyses were performed with PASW Statistics, version 18.0.0 (IBM, New York, USA).

## 3. Results

Of the 563 enrolled participants, 487 completed the study, 32 died, and 38 dropped out due to various disease states interfering with study follow-up or unwillingness to complete the study. Over the 3-year period, 68 participants experienced a CVD event of which 14 were fatal and 54 were nonfatal.

Baseline characteristics of the total population are shown in Table 1. For the whole cohort, median age was 70 years, 30% had hypertension, 28% established CVD, and 34% were current smokers. Except for age (69.6 years in the placebo group versus 70.4 years in the n-3 PUFA group; *P* = 0.013), there were no significant differences between the randomized treatment groups.

### 3.1. Effects of Intervention

**Figure 1:**
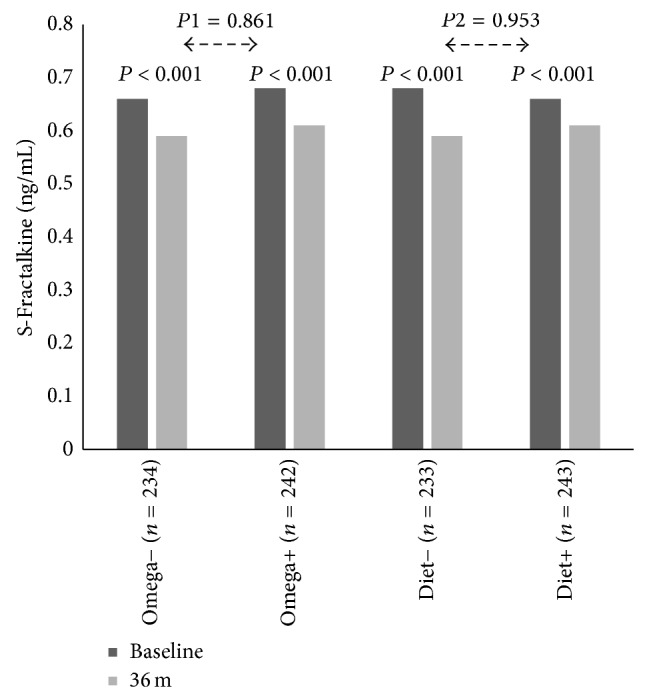
Serum fractalkine levels at baseline and after 36-month intervention according to the factorial design. *P* < 0.001 refers to difference within groups from baseline to 36 m,* P*1 value refers to difference in changes between O− and O+ from baseline to 36 m, and* P*2 value refers to difference in changes between D− and D+ from baseline to 36 m. Omega−: placebo capsules. Omega+: n-3 PUFA supplementation (capsules). Diet−: no diet intervention. Diet+: Mediterranean-like diet intervention.

Treatment effects on serum lipids have previously been reported, showing a significant reduction in triglycerides in the n-3 PUFA group [27]. Results from food frequency questionnaires and fatty acid analysis in a random subset of patients (*n* = 278) at baseline and after 36 months indicate satisfactory compliance with intervention [27].  Figure 1 shows the treatment effect of Mediterranean-like diet and/or n-3 PUFA supplementation on circulating levels of fractalkine analyzed according to the factorial design. Fractalkine levels were significantly reduced from baseline to 3 years in all intervention groups (*P* < 0.001). There were, however, no between-group differences in changes. No correlation between the changes in fractalkine levels and changes in serum triglycerides (*r* = 0.047; *P* = 0.32), EPA (*r* = −0.070; *P* = 0.30), or DHA (*r* = 0.002; *P* = 0.98) was found in the total cohort. Looking specifically at the subgroup treated with both n-3 PUFA capsules and diet intervention (*n* = 115), which had the greatest reduction in triglycerides after 36 months, there was no difference in changes in fractalkine compared to the other subgroups combined (*P* = 0.45).

### 3.2. Prediction of Clinical Events

**Figure 2:**
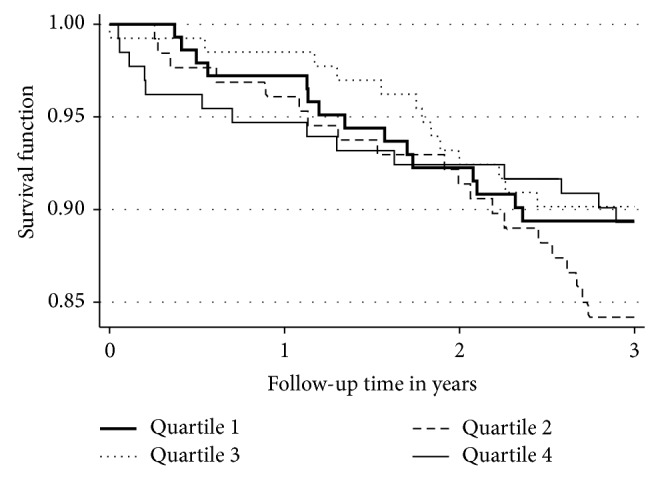
Kaplan-Meier plot: 3-year CVD events stratified by quartiles of fractalkine (*n* = 68).

We found no significant differences in changes of serum fractalkine levels between those who experienced a nonfatal CVD event (*n* = 54) and the rest of the cohort during the 3-year follow-up.  Figure 2 shows a Kaplan-Meier plot for the 3-year CVD events stratified by quartiles of fractalkine. Cox regression was used to estimate the effect of fractalkine in quartiles on time to CVD event adjusted for age, smoking, and treatment modality. There was no interaction between smoking and fractalkine for CVD, death, or any event. The HR for quartile 2 versus quartile 1 was 1.56 (0.80–3.06; *P* = 0.19), quartile 3 versus quartile 1 was 1.04 (0.49–2.19; *P* = 0.93), and quartile 4 versus quartile 1 was 1.13 (0.54–2.35; *P* = 0.75). For overall mortality the HRs were 1.03 (0.4–2.67; *P* = 0.95), 0.85 (0.30–2.42; *P* = 0.77), and 1.20 (0.47–3.04; *P* = 0.70), respectively.

### 3.3. Association to Clinical Entities at Baseline

We found no differences in the baseline levels of fractalkine between groups defined by hypertension, diabetes, metabolic syndrome, or established CVD. However, significant lower fractalkine levels were observed in smokers compared to nonsmokers (0.64 versus 0.69 ng/mL, *P* = 0.019). Correlation analyses revealed no significant correlations between serum fractalkine and age or any serum lipids. There was also no correlation between fractalkine and EPA or DHA levels at baseline.

## 4. Discussion

The main finding in the present study was that 3-year intervention with Mediterranean-like diet and/or n-3 PUFA supplementation did not affect serum levels of fractalkine different from controls. There was, however, a significant reduction in all groups during follow-up. Fractalkine did not predict future CVD events or mortality in this elderly high-risk population. Any relation to age, serum lipids, diabetes, or metabolic syndrome could also not be found, whereas significantly lower levels were observed in smokers.

Results from the PREDIMED study point to a reduction in inflammatory markers and endothelial and monocytic adhesion molecules after short-term Mediterranean diet intervention compared to low-fat diet control [25]. Interestingly, a polyphenolic compound present in grapes and red wine has shown suppressing effect on fractalkine expression on human endothelial cells [26]. In the Mediterranean-like diet group of our trial, no influence of dietary change on circulating fractalkine could be demonstrated. Patients in the subgroup acting as controls, receiving neither n3-PUFA nor Mediterranean-like diet intervention (*n* = 113), also had a significant reduction in circulating fractalkine after 36 months (*P* < 0.001), but with no difference in changes compared to the intervention groups. This counteracts the argument that n-3 PUFA and Mediterranean-like diet both possibly could affect fractalkine levels, and results would be masked in the factorial design of our study.

As no relation to age was found, one could speculate that the general reduction in serum fractalkine levels observed during follow-up was caused by changes in life style and behavior after the participants were included in the study on the criteria that they had high lipid levels and were at high risk of CVD.

The predictive value of soluble fractalkine on CVD could not be demonstrated in our population. It has been shown that a common genetic variant of the fractalkine receptor is independently associated with lower risk of CVD [30]. Also, soluble fractalkine was found to be an independent predictor of mortality in patients with advanced systolic heart failure, although the prognostic power did not differ between ischaemic and nonischaemic aetiology [13]. In the view of our investigation in a population of high-risk men, of whom 12% experienced a cardiovascular event, the predictive value of soluble fractalkine in atherosclerosis could be discussed.

Chapman et al. demonstrated that endogenous fractalkine cleaved from activated endothelial cell was not responsible for chemotaxis. They theorized that the release of fractalkine into soluble form does not really function as a chemotactic gradient for inflammatory cells, but rather that the cleavage of fractalkine from cell membrane is a “terminating event” reflecting a downregulation of its adhesion properties [31]. Umehara et al. also found fractalkine to function mainly as an adhesion molecule, with its soluble form having only a minor effect on monocytic cell line chemotaxis [32].

We found no association to clinical entities at baseline with regard to levels of serum fractalkine and also no significant correlations to age, lipids, or serum fatty acids. A recent large study by Xueyao et al. has shown a positive correlation between fractalkine and triglycerides and that fractalkine concentrations were associated with development of hypertriglyceridemia [14]. This could not be confirmed by our findings. As omega-3 fatty acids have documented triglyceride lowering effects [24], we were able to look at a subgroup of our population with a specially large reduction in triglycerides during the 3-year follow-up (mean triglycerides from 1,76 to 1,27 mmol/L) but no correlation with changes in serum fractalkine was found. This discrepancy is not easily explained, but Xueyaho et al. studied younger Chinese subjects at mean age of 57 years, without known CVD or treatment with lipid-lowering drugs. In our population triglyceride levels at baseline were almost 20% higher and 27% were using statins. It has been shown that statin therapy modifies the expression of fractalkine [12]; thus one could speculate that statins affect the association between fractalkine and triglycerides and that the association might only exist at normolipidemic levels.

We did observe significantly lower fractalkine levels in smokers compared to nonsmokers which is in accordance with previous result from a large cohort of CAD patients [15]. Rius et al. found that stimulation of human arterial umbilical endothelial cells with cigarette smoke extract induced expression of fractalkine and led to increased mononuclear cell adhesion [33]. They also noted that circulating monocytes and lymphocytes had upregulated fractalkine receptors in patients with chronic obstructive pulmonary disease. Since our findings are based on measured soluble fractalkine, it could be speculated that smokers heightened inflammatory state leads to a continuous expression of attached fractalkine on the cell surface; thus less is cleaved into circulating form. The smaller existing amount of soluble fractalkine could also then be affected by elevated binding to proinflammatory cell receptors (CX3CR1), in all, causing less amounts of measurable free fractalkine in serum.

There have been quite a few publications on the association of fractalkine with CVD, but there is a lack of larger clinical studies to validate these. Our study has limitations considering the same gender population, already at high risk of CVD and quite heavily medicated. As the initial trial was performed over a decade ago, current medical treatment regimes could have changed today, although the mainstay of treatment remains the same. The strengths of the study are attributed to the rather large population, the measure of compliance, and the long intervention period of 3 years.

## 5. Conclusion

Reduced levels of circulating fractalkine over a 3-year period were observed in elderly high-risk men and significantly lower levels were observed in smokers. However, intervention with Mediterranean-like diet and/or n-3 PUFA supplementation did not affect serum levels of fractalkine. Circulating fractalkine levels were not predictive for CV events or total mortality.

## Figures and Tables

**Table 1:** Baseline characteristics of the total study population (*n* = 563).

Age (y)	70.0 (67.5, 72.6)
Current smoker (%)	34
Body mass index (kg/m^2^)	26.5 (24.1, 28.7)
Metabolic syndrome (%)	39
Total cholesterol (mmol/L)	6.3 (5.7, 7.0)
LDL (mmol/L)	4.1 (3.5, 4.7)
HDL (mmol/L)	1.37 (1.15, 1.61)
Triglycerides (mmol/L)	1.53 (1.13, 2.04)
Fasting s-glucose (mmol/L)	5.6 (5.3, 6.2)
Systolic blood pressure (mmHg)	148 (135, 160)
Diastolic blood pressure (mmHg)	83.50 (91.00, 76.50)
Previous hypertension (%)	30
Previous diabetes (%)	15
Previous myocardial infarction (%)	18
Cardiovascular disease (%)	28
Aspirin (%)	26
Beta blocker (%)	17
ACE-I (%)	15
Calcium channel blocker (%)	16
Diuretic (%)	5
Nitrates (%)	9
Statins (%)	27

Data presented as percentages or median values (25, 75 percentiles).

ACE-I: angiotensin-converting enzyme inhibitors/angiotensin II receptor blockers; LDL: low density lipoprotein; HDL: high-density lipoprotein.
